# Co-existence of potentially sustainable indigenous food systems and poor nutritional status in Ho indigenous community, India: an exploratory study

**DOI:** 10.1088/1748-9326/ad4b44

**Published:** 2024-05-24

**Authors:** Ridhima Kapoor, Manisha Sabharwal, Suparna Ghosh-Jerath

**Affiliations:** 1 Department of Food and Nutrition and Food Technology, Lady Irwin College, University of Delhi-Sikandra Road, New Delhi 110001, India; 2 The George Institute for Global Health INDIA, New Delhi 110025, India

**Keywords:** indigenous food systems, indigenous communities, sustainable food systems, nutrition paradox

## Abstract

Indigenous food (IF) systems comprise culturally important foods derived from natural resources with shorter farm to plate chains, as compared to modern counterparts. These food systems are at epicenter of sustainable food systems with potential to alleviate malnutrition and protect ecosystems. The Ho indigenous community of Jharkhand have access to diverse IFs, yet experience malnutrition. A sequential mixed-method study was conducted to explore local food systems with assessment of diet quality and nutritional status of Ho women. Focus group discussions (*n* = 10) and free-listing (*n* = 7) were conducted to capture community’s perspectives on IF systems, associated sustainable attributes and perceived challenges towards IF utilization. Scientific names and nutritive values of IFs were documented through secondary review; in case of no information in literature, IFs were identified through ethnobotanist with nutritional analysis in laboratory. 24 h recalls (*n* = 284 in winter and *n* = 154 in monsoon) and anthropometric assessments (*n* = 273) were conducted to estimate women’s dietary diversity and nutritional status. Findings revealed rich traditional ecological knowledge (TEK) producing a diverse list of IFs (*n* = 243) mainly accessed from natural food environment (wild and cultivated). Among listed foods, 171 IFs were taxonomically classified and among these, nutritive values were documented for 121 IFs. Potentially sustainable traits of Ho’s IF systems included high nutrient density of IFs, community’s preference towards their consumption because of their flavorsome attributes, climate resilient properties and cultural heritage. However, impacts of climate variability, changing farming practices and market-integrated life ways challenged the sustained production, access and consumption of IFs. This was evident in poor diversity in diets (diet diversity scores of 2.9 in winter and monsoon) and chronic energy deficiency (36%) in women. The unique TEK systems of indigenous communities need to be integrated into mainstream policies and programs for safeguarding and promoting their potentially sustainable food systems to support food and nutritional security.

## 1 Introduction

The current global food systems are contributing to environmental degradation through unsustainable food production activities that are largely focused on cereal based mono-crops, making the global food plate less diverse [1–5]. These practices, along with the increasing nutritional needs of the growing population, challenge the capacity for sustainable healthy food production and consumption in the near future [5, 6]. All these issues have brought renewed attention towards the need to adopt a sustainable food systems approach using biodiverse underutilized foods to address global hunger, biodiversity loss and climate change [7].

Indigenous peoples are culturally and socially distinct groups who have resided in a region for thousands or hundreds of years, and retained their unique cultures and traditional ecological knowledge (TEK) and belief systems [8, 9]. These communities have unique food systems, which include all of the land, air, water, soil and culturally important plant, animal and fungi species that have sustained them over thousands of years [10]. Indigenous food (IF) systems comprise minor and/or endemic food crops (including native or underutilized species), farmer-saved varieties of major food staples, such as corn, rice, and wheat, as well as foods procured from local natural resources. These food systems are characterized by unprocessed foods, small-scale technology, with shorter farm to plate chains, as compared to their modern counterparts while preserving the local biodiversity [11– 14]. Therefore, IF systems are at the epicenter of sustainable food systems and may have potential in alleviating hunger, malnutrition and protecting the environment [15, 16].

Despite their potential importance towards ensuring nutritional security and environmental sustainability, the food systems of Indigenous Peoples are often perceived as ‘backward’ [17] and face threats due to impacts of industrialization and globalization of food systems, resulting in widespread losses of food-related biocultural diversity in many countries [18–20]. Further, due to impacts of poverty, marginalization and environmental degradation, Indigenous Peoples experience disproportionately high levels of malnutrition, maternal and infant mortality, and infectious diseases [21]. It is, therefore, crucial to better understand the relationship between Indigenous Peoples’ food systems and dietary outcomes to ensure effective and context-sensitive policies for food and nutritional security in these populations [18].

India is home to numerous indigenous communities (recognized as scheduled tribes or STs) [22] which make up a total population of 104 million [23]. Jharkhand, a central eastern state, is one of India’s most biodiversity-enriched regions and is home to 32 indigenous communities, that comprise more than one-fourth of the total state population [23]. Most indigenous communities in Jharkhand have unique food systems comprising several diverse species of edible and nutritious flora and fauna that are well-adapted to local natural ecosystems and hold special cultural significance for these groups [24–30]. Among the various indigenous groups in Jharkhand, the **‘Ho’** are one of the predominant communities that reside in biodiversity rich regions which are known to harbor rich and varied IFs [31, 32]. Despite having access to potentially nutrient rich IFs, indigenous communities of Jharkhand have high levels of maternal and childhood undernutrition [33–35].

The nutritional vulnerability of indigenous communities necessitates the need for exploring their IF systems to identify the different factors that contribute to a nutrition paradox. The present study aimed to explore the food systems of Ho community and investigate the diet quality and nutritional status of Ho women. Additionally, qualitative enquiries were used to contextualize the findings on diet and nutrition by identifying the interaction between various factors related to the food systems that may attribute towards nutritional outcomes in Ho community.

## 2 Methods

### 2.1 Study site

This study took place in West Singhbhum district (21°58’ to 23°60’ N, 85°0’ to 86°54’ E) of Jharkhand, in the administrative blocks of Sonua, Khuntpani and Chakradharpur. These regions have high population density of Ho tribal community [23]. The data collection sites included ten villages from the three blocks, randomly selected using probability proportional to size sampling.

### 2.2 Study design

A cross-sectional study design was implemented using a sequential mixed-method approach. This study design was chosen to first explore and document Ho community’s TEK, and perceptions around different attributes of their IF systems using qualitative methods. This was followed by associating these qualitative variables with quantitative outcomes such as dietary diversity and nutritional status of adult woman. Data was collected from September 2021 to August 2022, during the monsoon and winter seasons.

### 2.3 Sampling procedures

**Figure 1:**
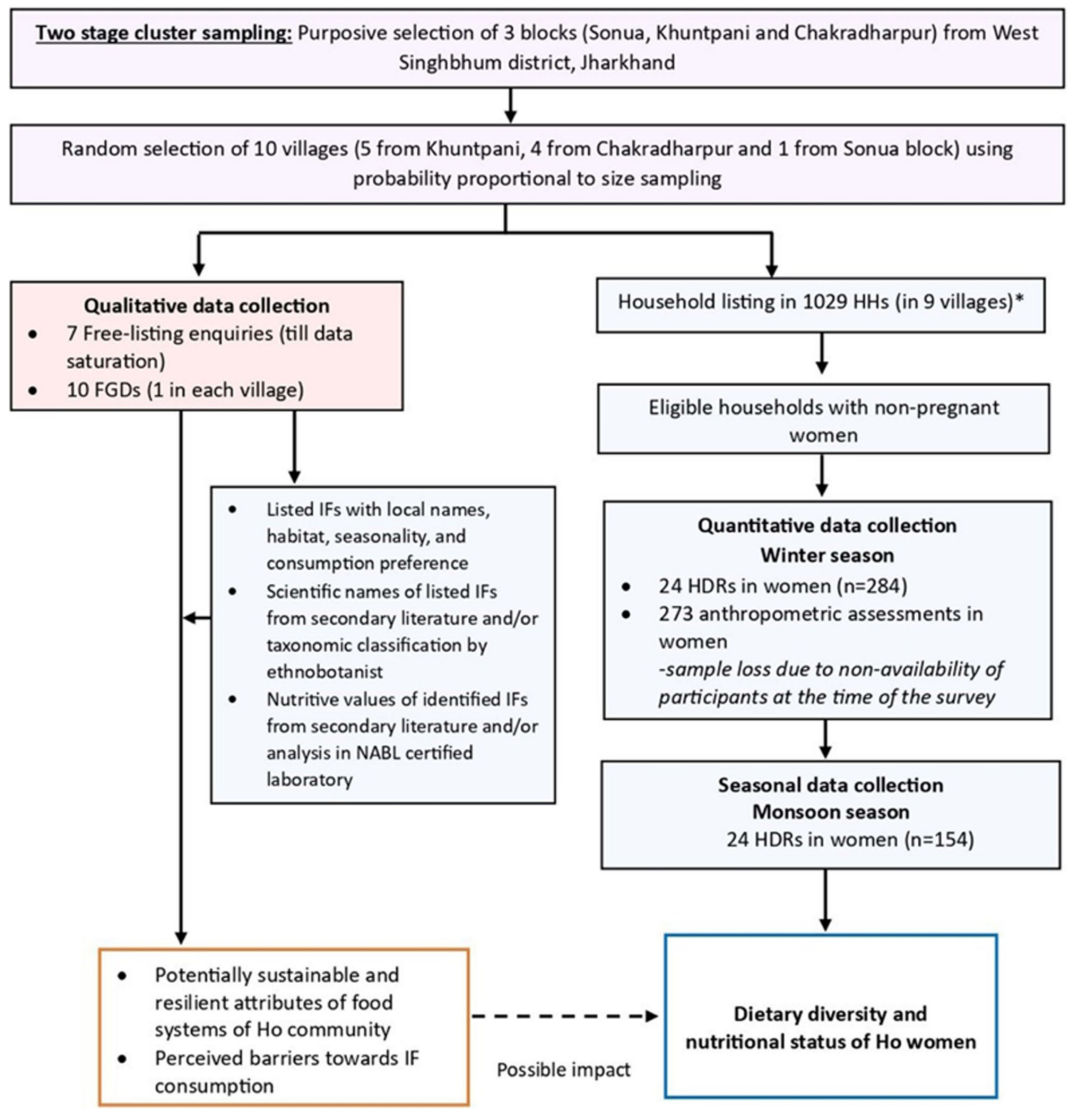
Study procedures and outcomes.

The present work was a part of a larger study that explored the contribution of IFs to food and nutritional security of indigenous communities in Jharkhand [36]. In this study, the sample selection for FGDs was done using snowball sampling [37]; wherein community health and nutrition workers in all study villages were asked to identify potential community members who were likely to have traditional knowledge on IFs, their accessibility, use and related information. Women were particularly encouraged to participate due to their primary involvement in household food collection, production and preparation [25]. For quantitative data collection, a total of 268 women were required to be sampled. This sample size was based on the difference (3.4 mg) in mean iron intake (SD of 7 mg d^*−*1^) among consumers and non-consumers of IFs in Santhal indigenous community of Jharkhand [38], using 80% statistical power based on a two-sided test having 5% level of significance. A design effect of 2 was assumed to account for the loss in precision due to cluster sampling. To achieve this sample size, a household listing was conducted in nine selected study villages (Sonua block was excluded due to safety issues for quantitative surveys) to generate a list of eligible households (presence of non-pregnant woman aged 18–49 years and under-five child-inclusion criteria for the larger study) (figure 1). Among the eligible households, dietary assessments and anthropometric surveys were completed in 284 women and 273 women respectively during the winter season. Seasonal dietary surveys were completed in 50% of the women (*n* = 154) surveyed during the monsoon season.

### 2.4 Data collection tools

#### 2.4.1 Qualitative enquiries

A PRA approach was used to rapidly characterize the IF systems of Hos, using structured FGD guides. A total of ten FGDs were conducted with a diverse group of community members (*n* = 50) including elders, women, men, and adolescent girls, who offered unique perspectives on their local food systems and their sustainable attributes. Topics included natural resource use, agricultural practices, food collection and hunting, cultural norms around IFs, consumption preferences and climatic changes. In our study, we defined sustainable food systems as those that contribute to food and nutrition security, and are culturally acceptable, while being protective of biodiversity and ecosystems [18, 39]. Free-listing, a qualitative data collection technique used to enlist all elements within a given domain [40], was also conducted to explore the community’s TEK regarding their IFs. These enquiries were conducted in seven villages with women (*n* = 41) to elucidate all the IFs known to Ho community, with details on their seasonality, source of access and frequency of consumption (commonly consumed/little consumed/historically consumed). All sessions were audio recorded.

#### 2.4.2 Taxonomic identification and nutritive values of IFs

Using secondary literature review, the scientific names and nutritive values of listed IFs were collated. In case of lack of information, the IFs were taxonomically identified through an ethnobotanist and the nutritive analysis was done at a NABL certified laboratory. A detailed methodology is provided in Supplementary Methods.

#### 2.4.3 Multipass 24-hour recalls

A non-consecutive, 2 d multipass 24 h dietary recalls (24HDR) [41, 42] were conducted by trained nutritionists to capture the dietary diversity of women from each selected household. In this method, women were prompted to provide detailed information on actual food and beverages consumed during the previous day, from waking up till retiring. A culturally appropriate, pictorial flipbook along with standard measuring cups and spoons were used to estimate portion sizes of raw and packaged foods.

#### 2.4.4 Anthropometry

Anthropometric assessments included weight and height measurements of same women approached for 24HDRs to measure their body mass index (BMI). These measurements were carried out using standard protocols [43] and equipment [weighing scale (Seca Model 813) and stadiometer (Seca Model 213)].

#### 2.4.5 Data analysis

All the FGDs were recorded and transcribed from *Ho* to Hindi and then translated to English. Information from the transcripts were used to develop a systematic list of IFs. Atlas.ti version 8 was used for coding the content of the transcripts, which was analyzed further using thematic analysis [44]. Common themes were generated which were compiled to summarize the sustainable attributes and challenges associated with local food systems. The quantitative variables were analyzed using descriptive statistics, which included computation of frequency counts and percentages for categorical variables, and mean and standard deviation for continuous variables Using 24HDR data, minimum dietary diversity score for women (MDD-W) was calculated, which identified the proportion of women (aged 15–49) who consumed at least 15 g of food from at least five of the ten food groups [45]. Based on this index, mean dietary diversity score (DDS) was computed by summing the total number of food groups consumed by the woman as reported in 2 days 24 HDR. BMI values [weight (in kg)/height (in m^2^)] were used to classify the nutritional status of women using standard cut-offs for Asian women [46].

#### 2.4.6 Ethical considerations

The study was conducted according to guidelines specified in Declaration of Helsinki [47] and ethical approval was obtained from Indian Institute of Public Health-Delhi, Public Health Foundation of India (Ref no: IIPHD_IEC_03_2017) and Lady Irwin College, University of Delhi (Ref no: Not available). Written administrative approvals from district authorities and cluster level consent from village leaders were obtained prior to data collection. For FGDs and quantitative surveys, a written consent was taken from literate participants and a verbal witnessed consent was sought from illiterate participants.

## 3 Results

The Hos are smallholder subsistence farmers residing in regions surrounded with forests and natural water bodies. They practice settled agriculture on plain farmlands and also forage foods from wild habitats for supplementing their diets.

The subsequent sections provide information on systematic documentation of their IF systems, their sustainable attributes, followed by quantitative estimates on diet quality and nutritional status of women. Finally, potential barriers towards IF consumption are discussed.

### 3.1 IF systems of Ho community

**Figure 2:**
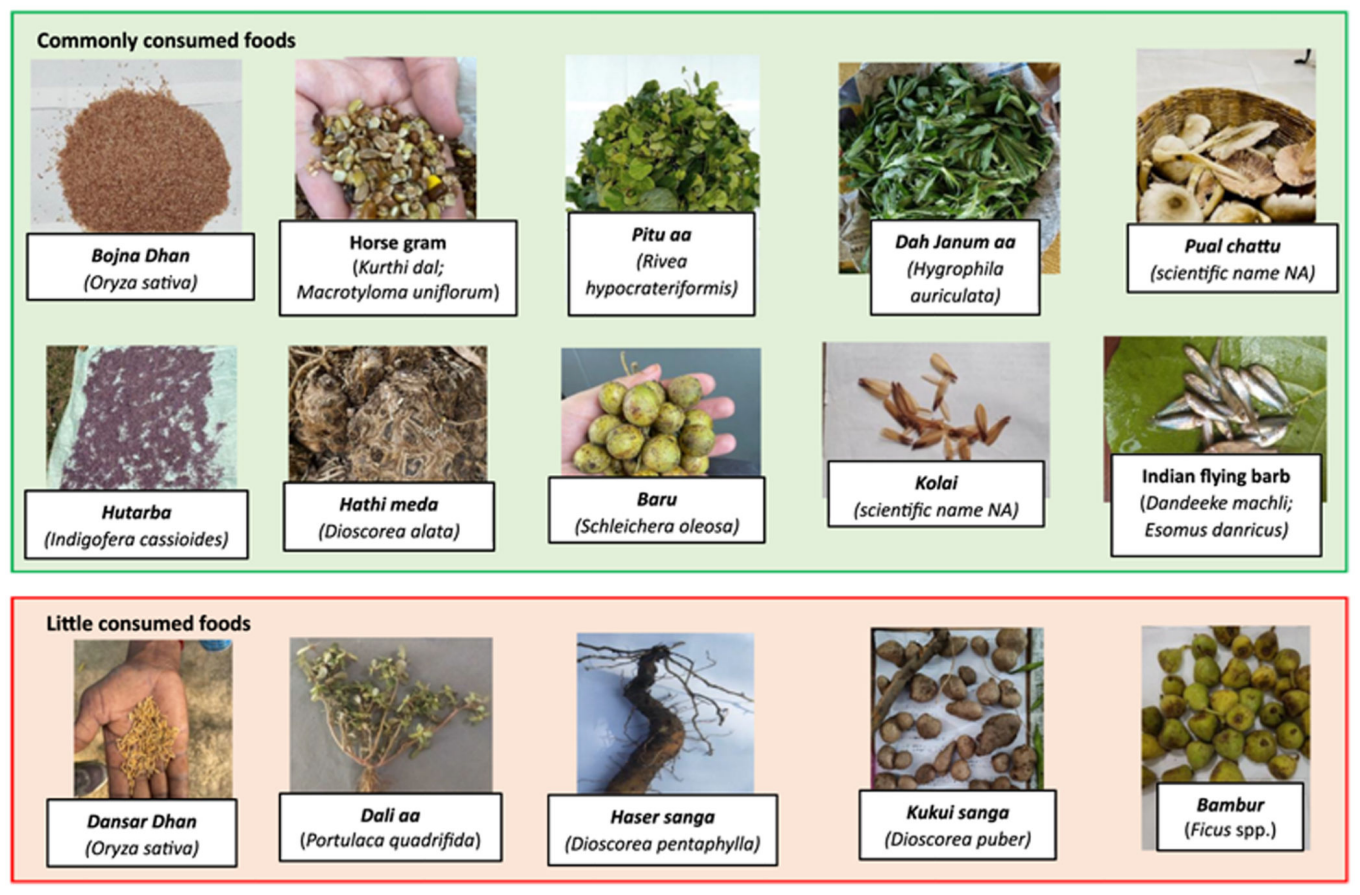
Indigenous foods of Ho tribal community of Jharkhand, India.

The community reported a vast TEK associated with their IF systems, which produced a diverse list of 243 IFs including 30 cereals (12%), 10 pulses (4%), 44 green leafy vegetables (GLVs) (18%), 51 other vegetables (including 34 mushrooms) (21%), 12 roots and tubers (5%), 21 fruits (9%), 73 flesh foods (meat, poultry, and fish) (30%) and 2 nuts and oilseeds (1%). The detailed list of IFs, their local names, scientific names, place of procurement, seasonality and preference of consumption are provided in Supplementary Table 1. Most foods accessed by Hos are seasonal, particularly fruits, GLVs and other vegetables (including wild mushrooms), with a majority (55.2%, *n* = 63) available during the monsoon season. Several GLVs, mushrooms, fruits, tubers, fishes and wild game are sourced from the wild food environment (forests, water bodies, roadsides and wastelands) while indigenous paddy varieties, pulses, vegetables and roots and tubers are obtained through cultivated food environment (farms and kitchen gardens). Some pictures of IFs are provided in figure 2.

**Figure 3:**
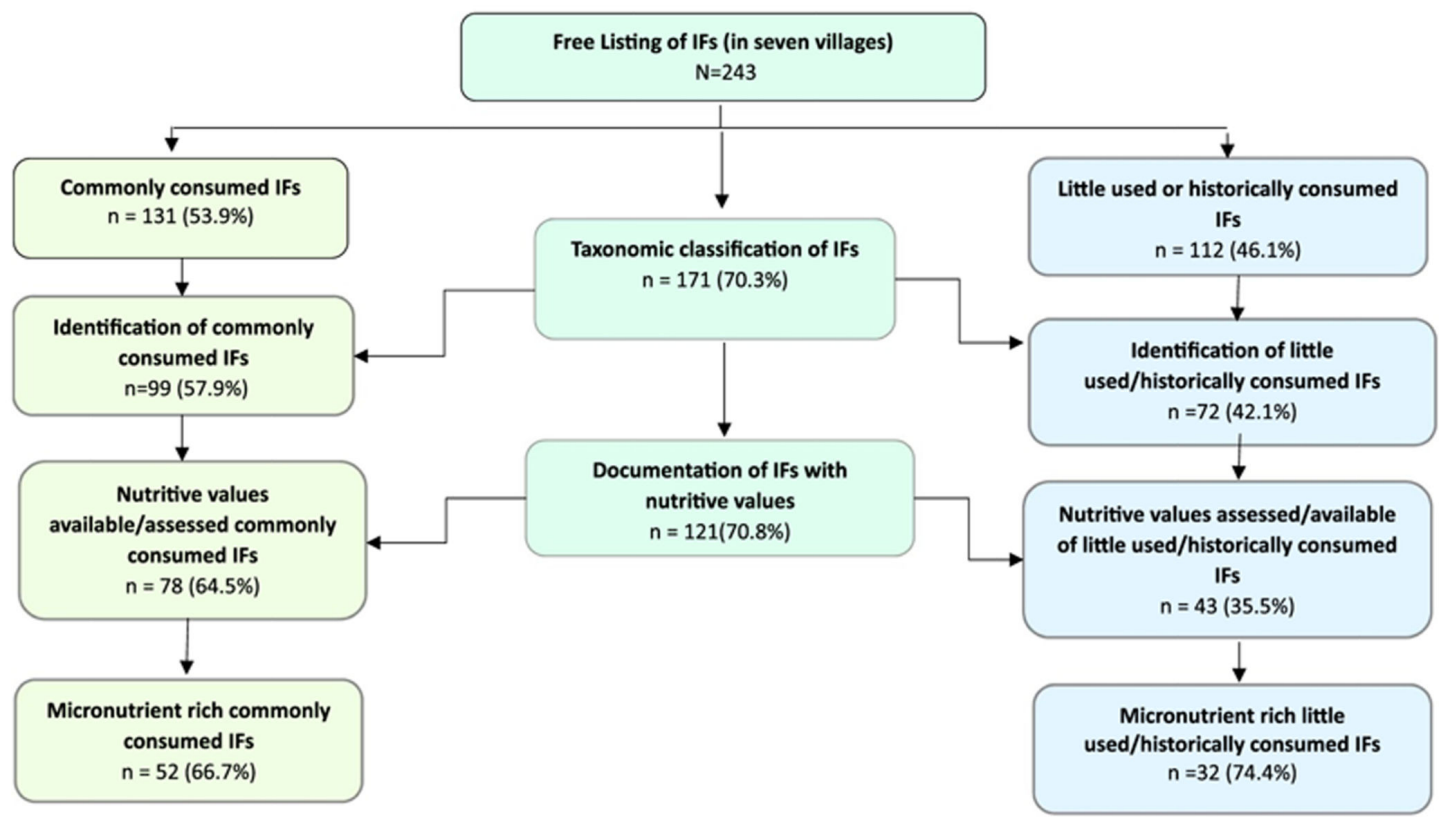
The systematic approach adopted to classify IFs available in Ho tribal community.

Based on the systematic documentation of scientific names and nutritive values, a majority of IFs (70%; *n* = 171) were taxonomically identified, with nutritive values documented for 121 IFs. Figure 3 summarizes the total number of identified IFs with nutritive values, under commonly consumed and little used/historically consumed categories. Among the several listed IFs, only 54% of the IFs were commonly consumed due to different factors discussed in the next section.

### 3.2 Potentially sustainable attributes of IF systems of Ho community

The Ho food system is diverse wherein foods are predominantly produced and accessed using traditional activities like farming, foraging, hunting, and fishing practices. Specific traits like nutrient density, climatic resilience and cultural heritage make these local food systems potentially sustainable, as highlighted below:

#### (i) Nutritionally superior IFs

Several IFs assessed by Hos were found to be rich in essential minerals and vitamins like iron, calcium, zinc, thiamine, folate, vitamin A and C (table 1). Specific indigenous varieties of rice, GLVs, fruits and flesh foods had higher nutrient density as compared to popular non-indigenous varieties. Indigenous GLVs like amaranth spined leaves (359 mg/100 g), bengal gram leaves (340 mg/100 g), garkha leaves (203 mg/100 g) were found to be good sources of calcium, as compared to mustard leaves (191 mg/100 g), and spinach (82.3 mg/100 g). In comparison to calcium content in fruits like ziziphus (46.5 mg/100 g), orange (19.5 mg/100 g), blackberry (23.8 mg/100 g), pineapple (10.8 mg/100 g), fig (78.5 mg/100 g), indigenous fruits like *bai* (364 mg/100 g), *mata* (138 mg/100 g) and *bambur* (128 mg/100 g)had exceptionally higher calcium content.

*Bojna dhan*, a commonly consumed indigenous rice variety, had higher iron content (7.8 mg/100 g) than conventional rice varieties (0.65 mg/100 g). Edible flowers of *Sanai* and *Hutarba*, locally consumed as vegetables, were good sources of iron (3.7–7.6 mg/100 g) and calcium (255–320 mg/100 g), as compared to popular vegetables like bitter gourd, bottle gourd, French beans brinjal, cauliflower and tomato respectively (iron range: 0.2–1.1 mg/100 g and calcium range: 8.9–49.9 mg/100 g) [48]. Additionally, wild roots and tubers were found to be rich in thiamine (range: 3.2–8.8 mg/100 g) and riboflavin content (range: 1.1–11.1 mg/100 g). These levels are much higher than the content reported in popular varieties like potato and colocasia (thiamine: 0.06 mg/100 g and riboflavin range: 0.01–0.03 mg/100 g). Routinely consumed insects like red ant and larvae of honeybee were exceptionally rich in iron (13.3–15.7 mg/100 g) and zinc (11.6–19 mg/100 g), much higher than the levels in conventional meats like poultry, beef, and pork (iron range: 1–1.9 mg/100 g and zinc range: 1.3–3.8 mg/100 g) [48].

#### (ii) Favorable cultural perceptions on IFs that increase their utilization

The flavorful attributes of IFs play an important role in their popularity and consumption in Ho community. Different varieties of **GLVs** (colocasia leaves, drumstick leaves, pot cassia (*Kayur aa*), bottle gourd leaves (*Suku aa*), water spinach (*Kalmi aa*)), wild mushrooms (*Busu ud, Muroom ud, Pata ud, Gitil* ud), **fruits** (kusum, hog plum, monkey jack *(Dahu*)) and **flesh foods** (quail red ant, *Pothi* fish) are mainly consumed for their flavorsome taste. The IFs are also valued for their satiety and health benefits, as claimed by one of the respondents: ‘*Even if we eat less quantity of “koyadhan”* (a rice variety), *stomach fills fast. Its starch water is also thick’* (male respondent, study village one, Chakradharpur block), while another respondent stated ‘*it is (indigenous food) helpful in reducing malnutrition…’* (male respondent, Keadchalam village, Khuntpani block). Apart from the perceived health and flavor attributes, strong cultural beliefs exist around IFs which facilitate their consumption during religious occasions and festivals. This includes consumption of unique dishes like lentils (M*asoor Dal*) prepared with dried indigenous fishes during festival of flowers (*Baha Parab*) and chapatis (bread) made from flour of indigenous *Arwa* rice, consumed for marking the end of sowing of paddy.

#### (iii) Indigenous crop production being low-resource intensive and climate resilient

The agricultural systems of Hos are considered adaptable to the local climate variability, terrain, and soil. Crops like underutilized local landraces of rice (*Dongor dhan* and *Goda dhan*), sponge gourd, ridge gourd, cowpea, field beans, drumstick leaves, *khesari* dal, flaxseeds, red amaranth leaves and ivy gourd were reported to thrive even when other crops failed to provide an assured yield. In addition to this, indigenous varieties of red gram, *khesari* dal, horse gram and field beans reportedly require minimal agricultural inputs in terms of manpower, fertilizer, and irrigation water.

### 3.3 Nutritional outcomes of Ho tribal women

#### 3.3.1 Dietary diversity

**Figure 4:**
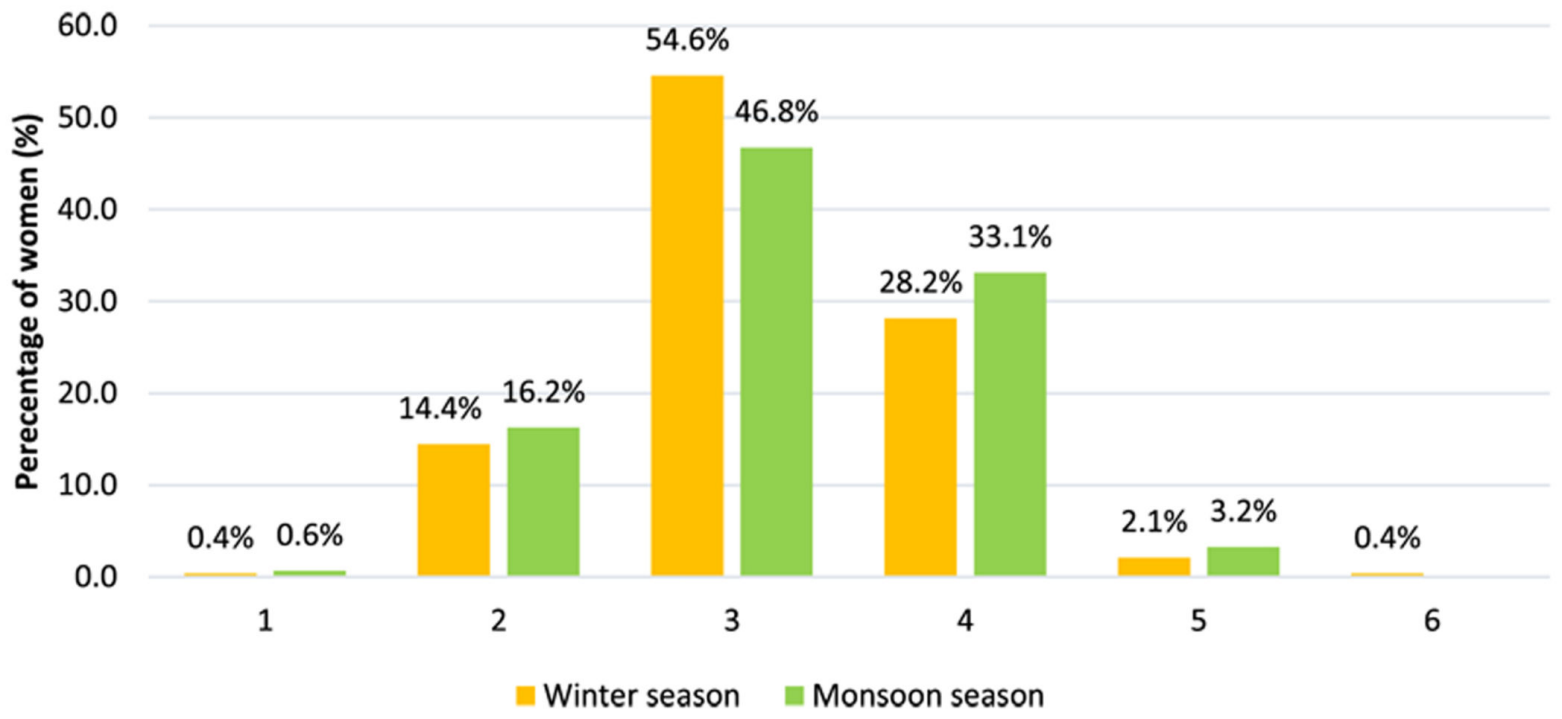
Distribution of DDS scores of Ho women across winter and monsoon seasons.

Most women consumed monotonous diets which primarily consisted of large amounts of starchy cereals (rice). Other foods consumed included small quantities of pulses, other vegetables (mostly onion and tomato as ingredients in dishes), and seasonal GLVs. Women consumed a carbohydrate-rich diet, with very low mean DDS in both seasons (2.9 *±* 1.4 in winter and 2.9 *±* 0.8 in monsoon season). Figure 4 presents the distribution of DDS of women across both seasons. Only a few women consumed an adequately diverse diet of five or more food groups (*n* = 6 in winters and *n* = 5 in monsoon). The strikingly low estimates of dietary diversity are concerning, especially in the presence of a wide range of IFs in their food environment as reported in qualitative enquiries.

#### 3.3.2 Nutritional status

**Figure 5:**
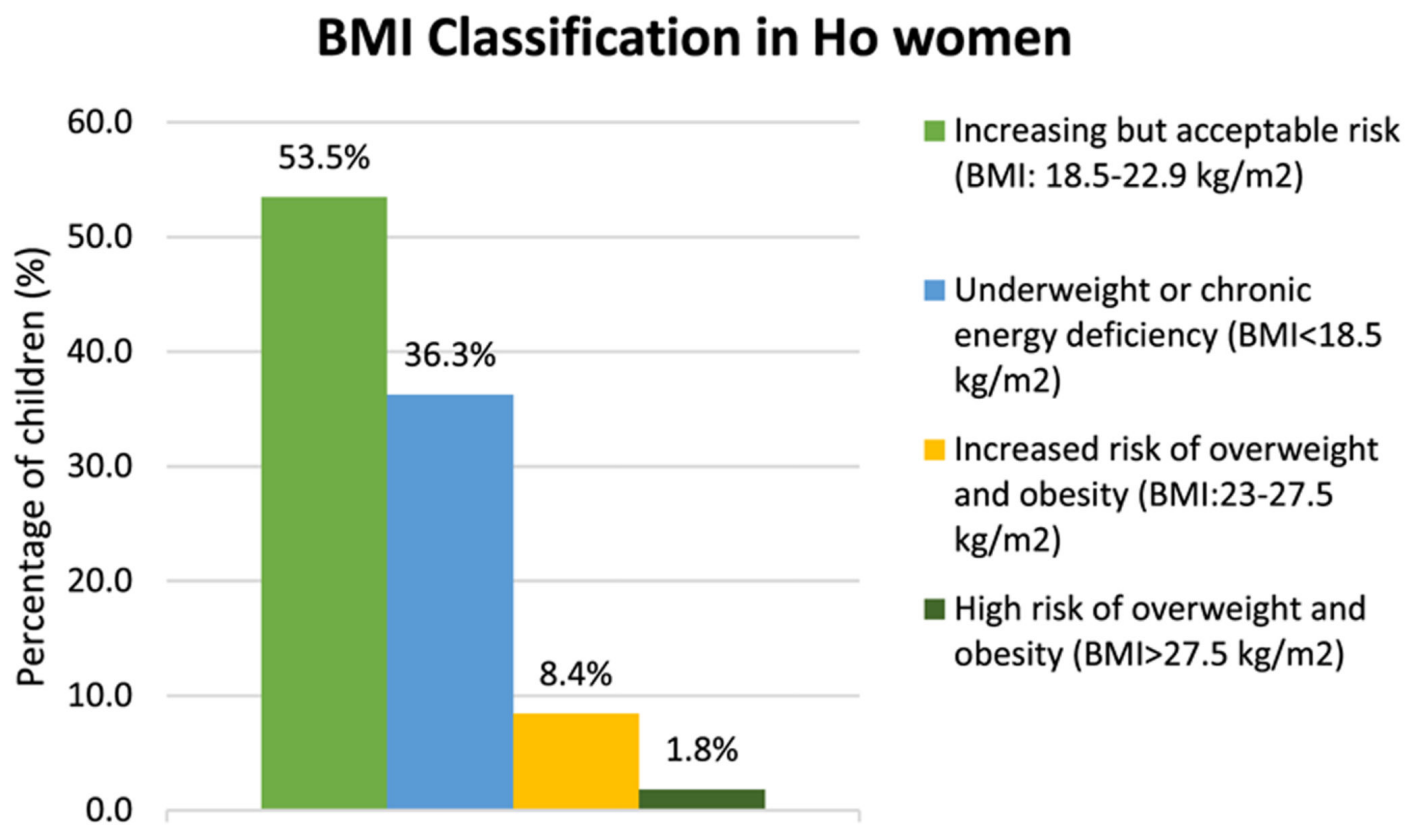
Nutritional status of Ho women.

The poor diet quality was also reflected in high levels of undernourishment in these women. About 36% women had varying degrees of chronic energy deficiency (CED) (BMI < 18.5 kg m^*−*2^), with nearly one-fourth in the category of CED III (<16 kg m^*−*2^) (figure 5).

### 3.4 Barriers towards effective utilization of IFs

**Figure 6:**
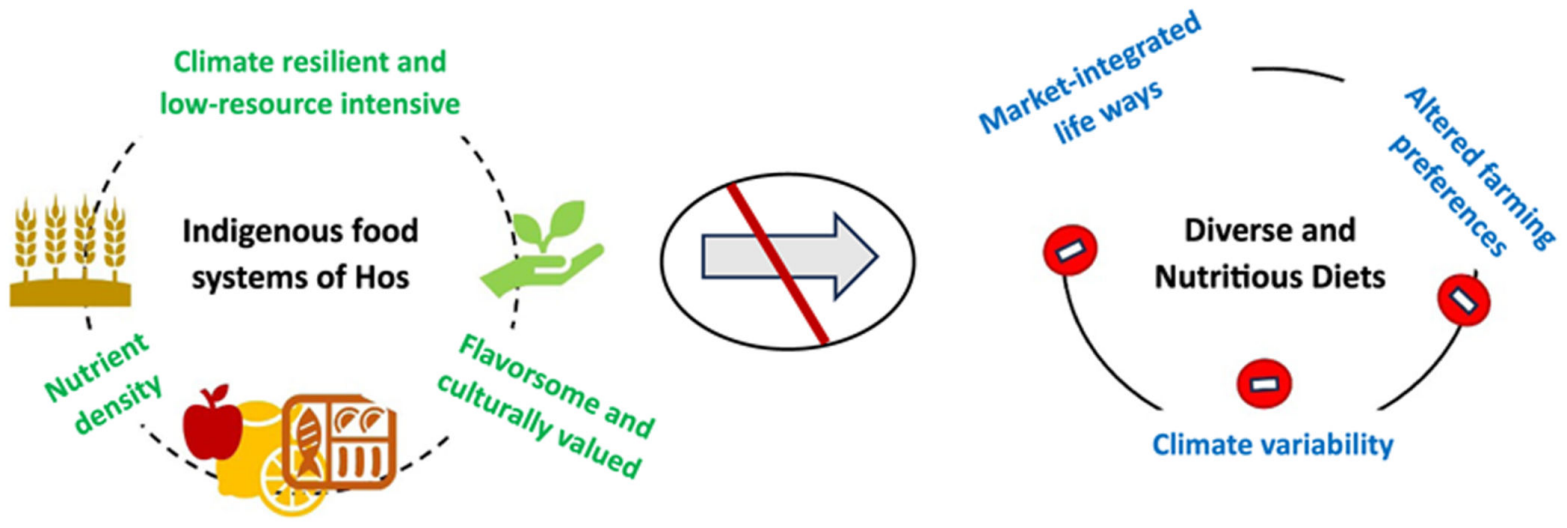
Nutrition paradox in Ho indigenous community of Jharkhand.

Despite a wide variety of micronutrient-rich IFs in the natural food environment of Ho community, women consumed poor quality diets and had varying levels of CED. This nutrition paradox is perhaps an outcome of interplay of diverse factors that potentially hinder the sustained production and access to IFs, thereby impacting their effective dietary utilization (figure 6). These barriers include (i) climate variability, (ii) altered farming preferences, and (iii) market integration resulting in shift from traditional to modern diets. Each of these barriers are detailed out below:

#### (i) Climate variability

The Ho IF systems are attuned to particular climates and ecosystems. Climate variability and change have disturbed this balance by impacting the ecosystems that support wild and domesticated edible plant and animal species. Since the past decade, these climatic variations are manifesting in the form of infrequent rainfall pattern. The delayed and untimely monsoon often lead to a shift in the sowing cycle leading to crop spoilage and poor yield. One person commented, ‘*It is not raining timely, so farming is also not happening on time. Earlier we used to sow paddy in April, but now it reaches even June*’ (female respondent, Bankitapi village, Chakradharpur block), while another person said ‘*This year (2021) we had heavy rain… and even on wrong timing*.. *Last year, we grew less paddy… this year also it is very less… even somepeopledidnotdofarming this year…*’ (female respondent, Keadchalam village, Khuntpani block). Since most farmers practise subsistence agriculture, the inadequate crop yield, especially for paddy, often creates food shortages within the household.

In order to cope with the climate impacts on their traditional farming systems, some farmers have switched from organic manure to chemical fertilizers for better crop yield, as stated by a women, ‘*We heard that earlier paddy used to be available in good quantity… heard about early times (around two decades back)… but now in our time, paddy is not growing well… in earlier time they used dung and compost for farming… nowadays fertilizers are being used… rain is not sufficient, so cultivation is not going well…’* (female respondent, Keadchalam village, Khuntpani block).

Further, the availability of wild forest foods like mushrooms (*Potkeh*), fruits (*Bai* and *Tova*) and wild tubers (*Kullu sanga* and *Piske sanga*) have greatly diminished due to irregular monsoon. In order to offset the low income from selling of insufficient farm and forest produce, some community members have started migrating in search of better earnings. Thus, the overall impact of climate-related changes have not only jeopardized the IF production and access, but have also altered the livelihood practices of Hos. The combined impact of these changes is leading to poor utilization of IFs, potentially resulting in monotonous diet.

#### (ii) Change in farming preferences

The current agricultural practices of Hos are predominantly based on paddy production along with cultivation of other food crops on farms and backyard gardens. Historically, the farming as well as the diets of Hos were not predominantly rice based. Around two decades back, the community used to cultivate crops like maize, sorghum, finger millet and niger seeds, which provided a diverse diet with potentially better nutritional quality. Owing to gradual loss of seeds of these crops, the community shifted to paddy cultivation. This has been one of the significant reasons for lack of diversity in the meals of Ho community. In the context of discontinued production of diverse crops, a village woman commented –’*Earlier (two decades back) we used to cultivate but now we do not find them here (seeds of local millets). Earlier we used to eat them but not now. Now we do not even cultivate it. It is like ‘mandua’ (ragi) which is not cultivated* (female respondent, Horlor village, Khuntpani block).

#### (iii) Market integration leading to a shift from traditional to modern diets

In the recent years, market penetration around the vicinity of study villages have created a dependence on built food environment provided by the markets. Although markets offer a potentially more diverse selection of foods, the gradual introduction of cheap ultra-processed foods and street foods in weekly markets or *Haats* are leading to a shift from traditional foods sourced from the natural food environment to calorie-dense, nutrient-poor, non-perishable highly processed foods accessed from built food environments.

Further, due to the market-integrated life ways, the Hos expressed foraging and hunting as time consuming activities. The access to forests (for some villages) owing to their distance from human habitats further add to this problem. One woman spoke: ‘*Yes, it is a hassle… even after going there (forest), we may not get it (foods)… sometimes we need to buy it (food) from market…’* (female respondent, study village three, Khuntpani block). The dependence on markets for habitual food consumption have been linked with decline in the foraging of certain indigenous varieties of **roots and tubers** like *Kukui sanga, Bayang sanga, Haser sanga*, ban-aloo (*Pitadu sanga*), **fruits** like Mata fruit (*Mata Soore*) and *Kandeyor*, **GLVs** like chimti leaves (*Mui aa*), phutkal leaves (*Phutkal saag*), beng leaves (*beng saag), Burnui aa* and **wild mushrooms** like *Neem poga, Sasang ud* and *Simdali ud*.

## 4 Discussion

The Ho indigenous community of Jharkhand reported a vast TEK on their IFs accessed from diverse food environments and comprising several diverse species of edible and nutritious flora and fauna. The local landraces were found to contain a wealth of micronutrients while being adapted to local climatic condition and geographical terrain. However, factors like climate variability, changing agricultural practices (with predominant focus on paddy production) and market-integrated life ways negatively impacted the availability and access of IFs in this community. Due to the diminished cultivation of indigenous varieties and poor access and utilization of several IFs, the usual meals of Hos lacked diversity and a significant proportion of women were undernourished with various grades of CED in the community. This complex interplay of barriers towards IF consumption and the poor nutritional outcomes could create a vicious cycle of diminished utilization of IFs, which may further erode the intergenerational TEK necessary for managing diverse and locally adapted food systems [53, 54].

In the recent decades, similar trends have been observed in different indigenous communities of India wherein a preferential use and promotion of cash crops have led to diminished cultivation of traditional food crops [55–58]. Further, monocropping (as observed in Ho tribe) may have negative implications on the environment, soil health and the survival of local landraces [59]. Similar to the present study observations, due to climate impacts, small-holder farmers across India and globally have resorted to different coping strategies such as adopting new crop varieties, incorporating modern farming practices, selling household assets, decreasing food consumption/changing diets and migration [28, 60, 61]. Further, in Ho community, the distal impacts of climate change on livelihood practices affected the household income, thereby potentially reducing affordability to diverse diets. This makes climate change, a significant issue for Indigenous Peoples, both in Indian and global context [62–64]. It is critical to involve Indigenous People in local, regional and national policies on climate change adaptation strategies which could not only strengthen their ability to better adapt to climate change impacts but also inform the local community about the local mitigation strategies [64].

In the present study, the perceived time-intensive nature of foraging was also an important barrier towards IF consumption. This has also been reported in other indigenous communities of India [24, 25, 32, 57, 58, 65, 66]. The reduced dependency on wild foods accompanied by a dietary shift towards convenience foods is a typical feature of nutrition transition that has become a common phenomenon among indigenous communities in India and globally [9, 25, 57, 67]. The ongoing ‘delocalization of food’ across indigenous communities undermines their food sovereignty and is leading to malnutrition of all forms in these populations [52, 68]. Thus, addressing this phenomenon through promotion of IFs is highly recommended.

Over several generations, Indigenous and tribal communities have evolved their TEK about lands, natural resources, and surroundings [18]. Relevant platforms to adopt, redefine and integrate these knowledge systems and mainstreaming them to policies and programs are critical in safeguarding these potentially sustainable food systems for improved nutrition in indigenous communities [69, 70]. Additionally, studies have shown that main-streaming neglected IFs into nutrition and agricultural policies and programs may revive IF systems and improve household income, food security, dietary diversity and micronutrient intake of women and children [71–73].

Although there is limited literature on Ho community’s perceptions around their IFs, particularly with respect to their consumption, studies on other indigenous communities of Jharkhand have also reported similar findings [24, 25]. Further, for Ho indigenous community, the existing nutrition data is available only in terms of anthropometric indicators (as part of district surveys). This study thus adds crucial information to the existing literature, by exploring the IF systems of one of the nutritionally vulnerable communities of India, and the contribution of these food systems towards diets and nutritional status of indigenous women.

## 5 Conclusion

The Ho indigenous community of Jharkhand have access to an abundance of nutritious and culturally acceptable IFs yet are consuming monotonous diets. Key interconnected factors related to climate, monocropping and increased proliferation of markets making energy-dense, nutrient-poor food commodities easily accessible, play a significant role in shaping up poor quality diets and high levels of under-nutrition in this community. However, a more in-depth analysis is required to ascertain the reasons for nutrition paradox in this community. Future research is needed to assess the impacts of different factors like climate indicators, soil health, socioeconomic drivers and women’s time use on Ho women’s dietary intake and nutritional status. Nonetheless, leveraging potentially sustainable traits of IF systems could offer opportunities to address undernutrition among the Hos. Safeguarding and promoting local food heritage should be a viable approach to strengthen food sov-ereignty and support nutrition and livelihood security in this community. Adequate institutional and policy support is needed to leverage the climate resilient traits of local landraces, which could further help in mitigating the climate impacts on food systems of Hos. Nutrition sensitive agricultural policies could also play a crucial role in promoting the use and conservation of neglected indigenous crops. A well-coordinated strategy that recognizes the unique relationship between indigenous culture and their foods, habitats, and ecosystems, could be a game changer in addressing the nutrition paradox in Ho community.

## Supplementary Material

Supplementary Materials

## Figures and Tables

**Table 1:** Nutritive values of some IFs accessed by Ho community.

S. No.	Food item (Local Name in Ho)	Common name (English/ Hindi)	Scientific name	Energy (Kcal/ 100 g)	Protein (g/100g	Carbo hydrate (g/ ) 100 g)	Fat (g/g)	Dietary fibre (g/ 100 g)	*β-* Caroten e/Retinol (*μ*g/ 100 g)	VitC (mg/ 100 g)	Vit B1 (mg/100 g)	Vit B2 (mg/100 g)	Total Folate (*μ*g/ 100 g)	Iron (mg/ 100 g)	Zinc (mg/ 100 g)	Calcium (mg/ 100 g)	Phosphorus (mg/ 100 g)	Vitamin D (g/100g)
1	*Koya dhan* ^b^	Rice	*Oryza sativa* L.	329	7.8	69.8	2.1	3.2	ND	ND	ND	ND	4	1.2	1.0	2.8	146.2	NA
2	*Bojno dhan* ^b^	Rice	*Oryza sativa* L.	335	8.1	70.0	2.6	3.5	ND	ND	ND	ND	3	7.8	0.8	6.2	169.6	NA
3	*Kanta saag* [48]	Amaranth spined leaves, green	*Amaran thus spinosus* L.	26	3.5	1.6	0.4	5.1	4174	82.6	0.0	0.1	41	6.3	1.6	359.0	72.4	15.2
4	*Sirgiti saag* [25]	GarkhaZ GadryaZ Garke	*Celosia argéntea* L.	48	4.6	6.4	0.4	5.9	1157	ND	ND	0.3	0	7.7	0.2	202.6	40.1	NA
5	*Garundi saag* [48]	Ponnaganni	*Alternanthera sessil is* (L.) R.Br. ex DC.	51	5.3	5.2	0.7	6.7	5288	103.0	0.0	0.1	48	3.9	1.0	388.0	58.3	0.7
6	*Chanasaag* [49]	Bengal gram leaves	*Cicer arietinum* L.	97	7.0	14.1	1.4	NA	NA	NA	NA	NA	NA	23.8	NA	340.0	120.0	NA
7	*Bambur* ^b^	—	*Ficus* spp.	38	4.5	5.0	ND	1.4	ND	ND	2.1	0.2	2	1.0	0.3	128.0	9.5	ND
8	*Baai* [49]	Banyan fruit	*Ficus benghalensis* L.	72	1.7	11.8	2.0	NA	NA	NA	NA	NA	NA	NA	NA	364.0	43	NA
9	*Matasoore* [49]	Mata fruit	*Antidesma acidum* Retz.	59	1.9	10.6	1.0	NA	NA	NA	NA	NA	NA	NA	NA	138.0	28.0	NA
10	*Hutarba* ^b^	—	*Indigofera cassioides* D.C.	73	5.3	12.9	ND	1.4	ND	ND	0.3	0.1	4	3.7	0.8	255.0	9.1	NA
11	*Sutri ba/sokoi* *sing* [25]	Sanai phool	*Crotalaria júncea* L.	120	2.9	27.0	ND	7.4	1112	1.8	3.1	ND	ND	7.6	0.2	320.2	537.2	NA
12	*Potkeh/* *Rotkeh/ Rugda* [25]	Mushroom	*Astraeus hygrometricus*	138	4.9	29.5	0.1	7.4	ND	ND	0.6	0.4	0	6.8	3.1	193.4	30.2	NA
13	*Kunyad ud* [25]	Mushroom	*Termitomyces*	41	2.5	7.5	0.2	6.1	9	ND	1.7	0.5	3	6.5	1.0	11.2	9.9	2.2
14	*Hadah/* *Oal/Pindi* [25]	Oal	*Amorphophallus* *paeoniifolius* (Dennst.) Nicolson	64	6.3	9.7	ND	1.2	ND	3.1	ND	ND	ND	11.1	1.1	35.7	45.0	NA
15	*Hathi Meda/* *Hathi manda* [25]	Khamaloo/ Chupri-aloo	*Dioscorea alata* L.	*126*	3.3	27.8	0.3	3.7	11	5.6	6.0	11.1	ND	3.8	0.4	10.7	28.1	NA
16	*Pitadu Sanga/* *Piske sanga* [25]	Ban-aloo/ Gethia kanda	*Dioscorea bulbifera* L.	44	2.1	9.0	ND	5.3	ND	ND	1.8	5.1	1	1.8	0.1	4.9	8.6	NA
17	*Kukui sanga* ^b^	—	*Dioscorea puber* Bl.	80	2.2	17.9	ND	2.1	ND	1.4	3.2	1.1	23	2.2	1.0	14.0	54.3	NA
18	*Kurkut/* *Hauko/* *Lal cheenti* [50]	Red ant	*Oecophylla* *smaragdina* Fabricius 1775	385	55.2	NA	14.9	NA	NA	NA	NA	NA	NA	15.7	19.0	74.7	NA	NA
19	*Kurkutanda* [49]	Eggs of red ants	*Oecophylla* *smaragdina* Fabricius 1775	131	13.4	9.1	4.6	NA	NA	NA	NA	NA	NA	NA	NA	104.0	107.0	NA
20	*Madhumakkhi* *(larvae)* [51]	Bee	*Apis mellifera* Linnaeus1758	456	35.3	46.1	14.5	NA	NA	NA	NA	NA	NA	13.3	11.6	84.9	782.5	NA
21	*Chirpi/* *Haad/* *Pita hayi* [49]	Puti fish/Pool barb	*Barbus* sp.	106	18.1	3.1	2.4	NA	NA	15.0	NA	NA	NA	1.0	NA	110.0	NA	NA
22	*Ghonga* [52]	Snail	*Pila globosa*	97	10.5	12.4	0.6	NA	NA	NA	NA	NA	NA	NA	NA	870.0	116.0	NA
23	*Koo cha Hayi* [52]	Freshwater Mud eel	*Monopterus cuchia* *(Ham,1822)*	92	18.7	2.4	0.8	NA	NA	NA	NA	NA	NA	NA	NA	185.0	119.0	NA
24	*Seep/Gechi* [50]	Oyster	*Crassostrea* *madrasensis*	NA	NA	NA	NA	NA	2^a^	0.5	NA	NA	NA	3.2	3.5	168.7	319.5	NA

NA= Not available; ND= Not detected.

a Retinol expressed in *µ*g/100 gm for animal foods.

b Laboratory analysis conducted as part of the study.

## Data Availability

The data cannot be made publicly available upon publication because no suitable repository exists for hosting data in this field of study. The data that support the findings of this study are available upon reasonable request from the authors.
