# Supplementary material for: Co-existence of potentially sustainable indigenous food systems and poor nutritional status in Ho indigenous community, India: an exploratory study
Source: Environ Res Lett. Author manuscript; Available in PMC 2025 Mar 25. (PMC7617527; doi:10.1088/1748-9326/ad4b44)
Supplement: Supplementary Materials [file EMS203906-supplement-Supplementary_Materials.pdf]

Supplementary Table 1: Indigenous foods, with taxonomic classification, part(s) consumed, seasonality, place of procurement, preference, and saliency

Local name	Common name	Scientific name	Part Consumed	Seasonality	Grown/Accessed	Food Preference
1. *Koya Dhan*	Variety of Rice	*Oryza sativa* L.[1]	Grain	Monsoon (sowing) and Winter (harvesting)	Field (Pi)	Commonly consumed
2. *Sorna Dhan*					Field (Sal)	Commonly consumed
3. *Bojna Dhan*					Field (Pi)	Commonly consumed
4. *Lal dhan/Arah dhan*					Field (Sal)	Little consumed
5. *Dudukulum Dhan*					Field (Pi), Kitchen Garden	Commonly consumed
6. *Goda Dhan*					Field (Goda)	Little consumed
7. *Jarli Dhan*					Field (Sal)	Commonly consumed
8. *Aril Dhan*					Field (Sal)	Little consumed
9. *Matkam Baba*					Field (Sal, Pi)	Historically consumed
10. *Sengel Baba*					Field (Sal)	Little consumed
11. *Hundukuya/Pundukuya Dhan*					Field (Sal)	Commonly consumed
12. *Kulugutu Dhan*					Field (Pi)	Commonly consumed
13. *Taisum Dhan*					Field (Pi)	Commonly consumed
14. *Kausum Dhan*					Field (Pi)	Little consumed
15. *Chini Baba*					Field (Pi)	Little consumed
16. *Dansar/Sardani Baba*					Field (Sal)	Little consumed
17. *Jemta Dhan*					Field (Pi)	Little consumed
18. *Marto Dhan*					Field (Sal)	Commonly consumed
19. *Jeera Baba*					Field (Sal)	Commonly consumed
20. *Dongor Baba*					Field (Pi)	Little consumed
21. *Chauli Gangai*	Sorghum	*Sorghum bicolor* (L.) Moench.[1]	Grain	Winter	Field (Goda)	Little consumed
22. *Hende Dhan*	Variety of Rice	*Oryza sativa* L.[1]	Grain	Monsoon (sowing) and Winter (harvesting)	Field (Sal)	Commonly consumed
23. *Aparom Baba*					Field (Pi)	Little consumed
24. *Tikur Baba*					Field (Pi)	Little consumed
25. *Bajra*	Pearl millet	*Pennisetum glaucum* (L.) R.Br[1]	Kernel	Winter	Market	Little consumed
26. *Syaai Dhan/Sae dhan*	Variety of Rice	*Oryza sativa* L.[1]			Field (Sal)	Historically consumed

Local name	Common name	Scientific name	Part Consumed	Seasonality	Grown/Accessed	Food Preference
27. *Diku Dhan*			Grain	Monsoon (sowing) and Winter (harvesting)	Field (Sal)	Historically consumed
28. *Chama Baba*					Field (Pi)	Historically consumed
29. *Rajamai Dhan*					Field (Pi)	Little consumed
30. *Tepaeh*	Variety of Maize	*Zea mays* L.[1]	Cob	Winter	Field (Goda), Kitchen garden	Little consumed
31. *Rehad*	Red gram pulse	*Cajanus cajan*[1]	Seeds	Summer	Field (Goda), Kitchen Garden	Commonly consumed
32. *Kansari*	Khesari dal	*Lathyrus sativus* L.[1]	Seeds	Summer	Field (Pi)	Commonly consumed
33. *Desi Moong*	Green gram dal	*Phaseolus aureus*[1]	Seeds	Monsoon	Field (Goda), Kitchen garden	Commonly consumed
34. *Desi Masoor*	Lentil dal	*Lens culinaris*[1]	Seeds	Spring	Field (Goda),	Commonly consumed
35. *Desi Urad/Biri Dal*	Black gram dal	*Vigna mungo* (L.) Hepper[2]	Seeds	Monsoon	Field (Goda), Kitchen Garden	Commonly consumed
36. *Kudti*	Horse gram	*Macrotyloma uniflorum* (Lam.) Verdc. [2]	Seeds	Winter	Field (Goda), Kitchen garden, Market	Commonly consumed
37. *Batura/Kalai Dal*	Munmuna	*Vicia hirsuta* (L.) Gray[2]	Seeds	Spring	Field (Sal)	Little consumed
38. *Desi Chana Dal*	Bengal gram dal	*Cicer arietinum* L. [1]	Seeds	Winter	Market, Field (Goda)	Little consumed
39. *Sutri Ramba*	Rice Bean	*Vigna umbellata* (Thumb.) Ohwi & H. Ohashi[2]	Seeds	Monsoon	Field (Goda), kitchen garden	Little consumed
40. *Ghangra*	Cowpea, white	*Vigna unguiculata* (L.) Walp. [3]	Seeds	Winter	Field (Goda), Kitchen garden	Little consumed
41. *Tisi/Unchi*	Flaxseed/linseed	*Linum usitatissimum*[1]	Seeds	Spring	Field (Goda, Pi)	Little consumed
42. *Surguja*	Niger seeds	*Guizotia abyssinical*[1]	Seeds	Winter	Field (Goda)	Historically consumed
43. *Kacchu saag/Saru aa*	Colocasia leaves	*Colocasia esculenta* (L.) Schott[2]	Leaves	Monsoon, Winter	Forest, Kitchen Garden, Field (Goda), open spaces	Commonly consumed

Local name	Common name	Scientific name	Part Consumed	Seasonality	Grown/Accessed	Food Preference
44. *Munga saag/Mulga aa*	Drumstick leaves	*Moringa oleifera* Lam.[1]	Leaves	Summer	Kitchen Garden	Commonly consumed
45. *Mata aa*	Mata leaves	*Antidesma acidum* Retz.[2]	Leaves	Winter	Forest	Little consumed
46. *Leper aa/bhaji saag*	Amaranth, tender, green	*Amaranthus retroflexus* L.[1]	Leaves	All seasons	Field (Goda), Kitchen Garden, Market, open space	Commonly consumed
47. *Sarli saag*	Katai leaves	*Meyna pubescens* (Kurz.) Robyns[2]	Leaves	All seasons	Forest	Little consumed
48. *Chakod saag/Kayur aa/Chakonda saag*	Pot cassia	*Senna obtusifolia* (L.) H.S. Irwin & Barneby[2]	Leaves	Summer	Kitchen Garden, open space	Commonly consumed
49. *Bayang aa*	Khatta saag	*Cissus auriculata* Roxb.[2]	Leaves	Summer	Forest	Commonly consumed
50. *Kecho aa/Khapra saag*	Punarnava	*Boerhaavia procumbens* Banks ex Roxb.[4]	Leaves	Monsoon	Kitchen garden, open spaces	Commonly consumed
51. *Mui ah/Cheenti saag*	Chimti leaves	*Polygonum plebeium* R.Br.[2]	Leaves	Spring and summer	Field (Sal), Open space	Little consumed
52. *Pudrum saag/Araah ipeel aa*	Gogu leaves, red stem	*Hibiscus sabdariffa* L.[2]	Leaves	Autumn	Field (Goda), Kitchen Garden	Commonly consumed
53. *Chatom aa*	Sunsuni leaves	*Marsilea minuta* L.[2]	Leaves	Monsoon	Field (Sal), open space	Commonly consumed
54. *Chana saag*	Bengal gram leaves	*Cicer arietinum* L.[2]	Leaves	Winter	Field (Goda, Pi)	Commonly consumed
55. *Kansari aa*	Khesari leaves	*Lathyrus sativus* L.[2]	Leaves	Winter	Field (Pi)	Commonly consumed
56. *Rimil Tundu saag*	Dheniani	*Olax scandens* Roxb.[2]	Leaves	Summer	Open spaces, Forest	Commonly consumed
57. *Pui aa*	Malabar spinach	Basella alba L.[1]	Leaves	Monsoon	Kitchen Garden	Commonly consumed
58. *Garundi aa*	Ponnaganni	*Alternanthera sessilis* (L.) R.Br. ex DC.[1]	Leaves	Monsoon	Field (Sal), open space	Commonly consumed
59. *Lal bhaji/Lal saag*	Amaranth leaves, red	*Amaranthus gangeticus* L.[1]	Leaves	All seasons	Field (Goda), Kitchen Garden, Market, open space	Commonly consumed

Local name	Common name	Scientific name	Part Consumed	Seasonality	Grown/Accessed	Food Preference
60. *Kohda saag/Kakarwa saag*	Ashgourd leaves	*Benincasa hispida* (Thunb.) Cogn. [2]	Leaves	Monsoon	Kitchen Garden	Commonly consumed
61. *Suku aa/Lauki/Kaddu aa*	Bottle gourd leaves	*Lagenaria siceraria* (Molina) Standl. [4]	Leaves	Monsoon	Field (Goda), Kitchen garden	Commonly consumed
62. *Burnui aa*	NA	*Leucas montana* Spreng. [4]	Leaves	Monsoon	Forest	Little consumed
63. *Beng aa*	Beng leaves	*Centella asiatica* (L.) Urb.[2]	Leaves	Monsoon	Field (Sal), open space	Little consumed
64. *Oae manda saag/ Chendein kata aa*	NA	*Merremia macrocalyx* (Ruiz & Pavon) O'Donell[13]	Leaves	Winter, Spring	Field (Goda) Kitchen Garden, Forest	Commonly consumed
65. *Sirgiti aa*	Garkha/ Gadrya/ Garke	*Celosia argentea* L.[2]	Leaves	Summer	Field (Goda)	Little consumed
66. *Kudmi/Kalmi aa*	Water spinach	*Ipomoea aquatica* Forssk.[2]	Leaves	Summer, Monsoon	Field (Sal, Pi), water bodies	Commonly consumed
67. *Chamta aa*	Cauliflower leaves	*Brassica oleracea* var. *botrytis*[1]	Leaves	Winter	Field (Goda)	Commonly consumed
68. *Dali aa*	Nunia leaves	*Portulaca quadrifida* L.[2]	Leaves	Summer	Field (Goda, Pi), Open spaces	Little consumed
69. *Aadi Sanga aa/Shakarkand saag*	Sweet potato leaves	*Ipomoea batatas* (L.) Lam.[2]	Leaves	Autumn, Summer	Field (Goda), Kitchen Garden	Commonly consumed
70. *Bilay moo aa*	NA	*Hemigraphis latebrosa* (B. Heyne ex Roth) Nees[4]	Leaves	Summer	Forest	Commonly consumed
71. *Pitu aa*	NA	*Rivea hypocrateriformis* Chois.[13]	Leaves	Winter	Forest	Commonly consumed
72. *Jayati saag*	NA	*Vicia sativa* L.[4]	Leaves	Monsoon	Open space	Commonly consumed
73. *Koinaari saag/Sing aa*	Koinaar leaves	*Bauhinia purpurea* L.[2]	Leaves	Summer	Kitchen Garden, Forest	Commonly consumed
74. *Dah Janum ah/Muchri saag*	NA	*Hygrophila auriculata* (Schumach.) Heine [4]	Leaves	Summer, Monsoon	Field (Sal), open space	Commonly consumed

Local name	Common name	Scientific name	Part Consumed	Seasonality	Grown/Accessed	Food Preference
75. *Kanta sag*	Amaranth spined leaves, green	*Amaranthus spinosus* L.[1]	Leaves	All seasons	Field (Goda), Kitchen Garden, Market, open space	Commonly consumed
76. *Hessa aa*	Peepal saag	*Ficus religiosa* (L.) [2]	Leaves	Summer	Open Spaces	Little consumed
77. *Aloo aa*	Potato leaves	*Solanum Tuberosum* L.[2]	Leaves	Spring, Summer	Field (Goda), Kitchen garden	Commonly consumed
78. *Hundupud aa/Undu pu aa*	Kena leaves	*Commelina benghalensis* L.[4]	Leaves	Summer, Monsoon	Kitchen Garden, Open space	Commonly consumed
79. *Tupi/Goma aa*	NA	*Leucas cephalotes* (Roth) Spreng. [4]	Leaves	Monsoon	Field (Goda), Kitchen garden, Open space	Little consumed
80. *Reedgedri saag*	NA	*Medicago denticulata* Willd. [4]	Leaves	Winter	Farm, Kitchen garden	Commonly consumed
81. *Phutkal saag*	Phutkal leaves	*Ficus virens* Aiton[2]	Leaves	Summer	Open space	Little consumed
82. *Pilli aa*	Neem leaves	*Azadirachta indica* A. Juss.[5]	Leaves	Summer	Open space	Commonly consumed
83. *Boroeh aa*	Kantha leaves	*Dentella repens* (L.) J.R. Forst. & G. Forst.[2]	Leaves	Spring	Field (Sal, Pi) Open space	Commonly consumed
84. *Chota Tupi aa*	NA	*Leucas aspera* (Willd.) Link [4]	Leaves	Monsoon	Field (Goda), open space	Little consumed
85. *Saaka saag*	NA	Apocynaceae[4]	Leaves	Summer	Open spaces, Forest	Commonly consumed
86. *Charmani aa*	Hurhura	*Cleome monophylla* L. [2]	Leaves	Monsoon	Kitchen garden	Little consumed
87. *Nenua /Pullu*	Sponge gourd	*Luffa aegyptiaca* Mill. [4]	Vegetable	Summer	Field (Goda), Kitchen garden	Commonly consumed
88. *Jhinga/Junni*	Ridge gourd (small)	*Luffa acutangula* (L.) Roxb. [4]	Vegetable	Summer, Monsoon	Kitchen garden	Commonly consumed
89. *Barbatti/Bodi*	Barbatti vegetable	*Vigna unguiculata* (L.) Walp. [4]	Vegetable	Winter	Kitchen Garden	Commonly consumed
90. *Kundri*	Ivy gourd	*Coccinia grandis* (L.) Voigt[1]	Vegetable	Monsoon	Field (Goda), Kitchen Garden	Commonly consumed
91. *Sidmi (broad)*	Field beans, tender, broad	*Vicia faba* L.[1]	Vegetable	Winter	Field (Goda), Kitchen Garden	Commonly consumed

Local name	Common name	Scientific name	Part Consumed	Seasonality	Grown/Accessed	Food Preference
92. *Pandeya/Kohda/Kakdu*	Ashgourd	*Benincasa hipsida* (Thunb.) Cogn. [4]	Vegetable	Winter	Field (Pi, Goda)	Little consumed
93. *Gamarphali/Chayipi/Malhan*	NA	NA	Vegetable	Summer	Field (Goda), Kitchen garden	Commonly consumed
94. *Sidmi (thin)*	Field beans, tender, lean	*Vicia faba* L.[1]	Vegetable	Winter	Field (Goda), Kitchen Garden	Commonly consumed
95. *Desi Karela*	Biiter gourd, jagged teeth ridges, short	*Momordica charantia* L. [1]	Vegetable	Monsoon	Kitchen garden, market	Commonly consumed
96. *Buru Kundri*	NA	*Solena amplexicaulis* (Lam.) Gandhi [4]	Vegetable	Monsoon	Forest	Little consumed
97. *Ipil/Epil (peel of the fruit)*	NA	*Hibiscus sabdariffa* L.[2]	Vegetable	Monsoon	Field (Goda)	Commonly consumed
98. *Heksa/Kheksa /Kakrol*	Spine gourd	*Momordica dioica* Roxb.[2]	Vegetable	Monsoon	Forest	Commonly consumed
99. *Anjed jo*	Kutma	*Solanum torvum* Swartz. [2]	Vegetable	Monsoon	Open spaces, Field (Pi, Goda), Forest	Commonly consumed
100. *Rayuba*	NA	NA	Vegetable	Summer	Forest	Little consumed
101. *Sutri Baa/Sokoi Sing*	Sonpu flower	*Crotalaria juncea* L. [2]	Flower	Winter	Field (Goda)	Little consumed
102. *Hutarba*	NA	*Indigofera cassioides* D.C. [2]	Flower	Spring	Forest	Commonly consumed
103. *Mulge Phool*	Drumstick flower	*Moringa oleifera* Lam.[1]	Flower	Winter	Open spaces	Commonly consumed
104. *Pata ud*	NA	NA	Fruiting body of fungi	Monsoon	Forest	Commonly consumed
105. *Potkeh/Rotkeh/ Rugda*	NA	*Astraeus hygrometricus*[12]	Fruiting body of fungi	Monsoon	Forest	Little consumed
106. *Idir ud/Bor ud*	NA	*Termitomyces albuminosa*[2]	Fruiting body of fungi	Monsoon	Forest, Kitchen Garden, Open spaces	Little consumed
107. *Gitil ud*	NA	NA	Fruiting body of fungi	Monsoon	Forest, Market	Commonly consumed
108. *Pual chattu/Busub ud*	NA	NA	Fruiting body of fungi	Monsoon	Forest	Commonly consumed

Local name	Common name	Scientific name	Part Consumed	Seasonality	Grown/Accessed	Food Preference
109.*Gein*	NA	NA	Fruiting body of fungi	Monsoon	Forest	Little consumed
110.*Porob ud/Indi ud/Dushara ud*	NA	NA	Fruiting body of fungi	Monsoon	Forest	Little consumed
111.*Kadaye/Kadhai ud*	NA	NA	Fruiting body of fungi	Monsoon	Open Spaces	Little consumed
112.*Muroom ud/Hende ud*	NA	NA	Fruiting body of fungi	Monsoon	Forest	Commonly consumed
113.*Simdali ud/Rang Birange Pata ud*	NA	NA	Fruiting body of fungi	Monsoon	Forest	Little consumed
114.*Angar ud*	NA	NA	Fruiting body of fungi	Monsoon	Forest	Commonly consumed
115.*Sasang ud*	NA	NA	Fruiting body of fungi	Monsoon	Forest	Little consumed
116.*Eede ud*	NA	NA	Fruiting body of fungi	Monsoon	Forest	Commonly consumed
117.*Bunum ud*	NA	NA	Fruiting body of fungi	Monsoon	Forest, Open Spaces	Little consumed
118.*Patka ud*	NA	NA	Fruiting body of fungi	Monsoon	Forest	Little consumed
119.*Sosoye ud*	NA	NA	Fruiting body of fungi	Monsoon	Forest	Commonly consumed
120.*Cheeru ud*	NA	NA	Fruiting body of fungi	Monsoon	Forest	Little consumed
121.*Ude ud*	NA	NA	Fruiting body of fungi	Monsoon	Forest	Commonly consumed
122.*Tumbe ud*	NA	*Pisolithus arhizus* (Scop.) Rauschert[8,9]	Fruiting body of fungi	Monsoon	Open Spaces	Commonly consumed
123.*Aatta ud/Adaa ud*	NA	*Termitomyces reticulates* Van der Westh. & Eicker[9]	Fruiting body of fungi	Monsoon	Forest	Commonly consumed
124.*Hashangar ud*	NA	NA	Fruiting body of fungi	Monsoon	Forest	Little consumed

Local name	Common name	Scientific name	Part Consumed	Seasonality	Grown/Accessed	Food Preference
125.*Bair poga*	NA	NA	Fruiting body of fungi	Monsoon	Open Spaces	Little consumed
126.*Saaga ud*	NA	NA	Fruiting body of fungi	Monsoon	Forest	Commonly consumed
127.*Kunyad ud*	NA	*Termitomyces*[2]	Fruiting body of fungi	Monsoon	Forest	Commonly consumed
128.*Surjum ud/Sal poga*	NA	NA	Fruiting body of fungi	Monsoon	Forest	Historically consumed
129.*Neem poga*	NA	NA	Fruiting body of fungi	Monsoon	Open Spaces	Little consumed
130.*Paiteh ud*	NA	NA	Fruiting body of fungi	Monsoon	Open Spaces	Commonly consumed
131.*Aam poga*	NA	NA	Fruiting body of fungi	Monsoon	Open Spaces	Little consumed
132.*Hathi Manda ud*	NA	*Boletus edulis*[2]	Fruiting body of fungi	Monsoon	Forest	Commonly consumed
133.*Daru poga ud*	NA	NA	Fruiting body of fungi	Monsoon	Forest	Historically consumed
134.*Chimboor*	NA	NA	Fruiting body of fungi	Monsoon	Forest	Commonly consumed
135.*Loyong ud*	NA	NA	Fruiting body of fungi	Monsoon	Forest	Commonly consumed
136.*Uju ud*	NA	NA	Fruiting body of fungi	Monsoon	Forest	Commonly consumed
137.*Koode ud*	NA	NA	Fruiting body of fungi	Monsoon	Forest	Historically consumed
138.*Pitadu Sanga/Piske sanga*	Ban-aloo/ Gethia kanda	*Dioscorea bulbifera* L.[4]	Tuber	Winter	Field (Goda)	Little consumed
139.*Hadah/Oal/Pindi*	Desi Oal	*Amorphophallus paeoniifolius* (Dennst.) Nicolson[2]	Tuber	Monsoon	Field (Goda), Kitchen garden	Commonly consumed
140.*Hathi Meda/Hathi manda/Roa mundu sanga*	Khamaloo/ Chupri-aloo	*Dioscorea alata* L.[4]	Tuber	Winter	Kitchen garden	Commonly consumed

Local name	Common name	Scientific name	Part Consumed	Seasonality	Grown/Accessed	Food Preference
141.*Bayang sanga*	NA	*Dioscorea glabra* Roxb.[6]	Tuber	Autumn	Forest	Historically consumed
142.*Haser sanga*	NA	*Dioscorea pentaphylla* L.[4]	Tuber	Autumn	Forest	Historically consumed
143.*Merom Tova/Cholo sanga*	NA	NA	Tuber	Winter	Kitchen garden	Commonly consumed
144.*Kukui sanga*	NA	*Dioscorea puber* Bl.[4]	Tuber	Winter	Forest	Little consumed
145.*Kullu sanga*	NA	*Dioscorea hispida* Dennst.[6]	Tuber	Monsoon	Forest	Little consumed
146.*Duri Sanga*	NA	*Dioscorea belophylla* Voight[4]	Tuber	Monsoon	Forest	Little consumed
147.*Unu Sanga*	NA	*Discorea* sp.[4]	Tuber	Autumn	Forest	Little consumed
148.*Kesari sanga*	NA	*Schoenoplectus grossus* (L.f) Palla [Cyperaceae][6]	Tuber	Autumn	Marshy land	Commonly consumed
149.*Kundri Sanga*	NA	*Solena amplexicaulis* (Lam.) Gandhi[4]	Tuber	Monsoon	Forest	Historically consumed
150.*Baru*	Kusum fruit	*Schleichera oleosa* (Lour.) Merr.[2]	Fruit	Monsoon	Trees (village, open space)	Commonly consumed
151.*Kendu/Tiril*	Tumki	*Diospyros melanoxylon* Roxb.[2]	Fruit	Summer	Forest	Commonly consumed
152.*Chahar/Chironji/Tarop*	Char/Piar	*Buchanania lanzan*[2]	Fruit	Summer	Forest	Commonly consumed
153.*Burui*	NA	NA	Fruit	Winter	Forest	Commonly consumed
154.*Daau/Dahu*	Barhar	*Artocarpus lakoocha* Roxb.[2]	Fruit	Summer	Trees, Open space	Commonly consumed
155.*Aain jo/Anjeer/Gular/Loa*	Gular	*Ficus racemosa* L.[2]	Fruit	Summer	Open space	Little consumed
156.*Amda/Ambau ba*	Hog plum	*Spondias Pinnata* (L.f.) Kurz.[2]	Fruit	Monsoon	Kitchen Garden, Forest	Commonly consumed
157.*Kodom*	NA	*Neolamarckia cadamba* (Roxb.) Bosser[4]	Fruit	Spring	Forest, Open field	Commonly consumed
158.*Baai*	Banayan Fruit	*Ficus benghalensis* L.[2]	Fruit	Summer	Forest	Little consumed

Local name	Common name	Scientific name	Part Consumed	Seasonality	Grown/Accessed	Food Preference
159.*Bambur*	NA	*Ficus* spp. [4]	Fruit	Winter	Forest	Little consumed
160.*Soso*	Marking nut (kernel)/ Bhelwa	*Semecarpus* anacardium L.f. [1]	Fruit	Winter	Forest	Little consumed
161.*Papah*	Paprah	*Gardenia latifolia* Aiton. [4]	Fruit	Summer	Forest	Little consumed
162.*Mata soore*	Mata fruit	*Antidesma acidum* Retz. [2]	Fruit	Monsoon	Forest	Little consumed
163.*Mirle/Pindra*	NA	*Flacourita indica* (Burm f.) Merr. [7]	Fruit	Monsoon	Forest	Commonly consumed
164.*Ranaba*	NA	NA	Fruit	Winter	Forest	Commonly consumed
165.*Bel*	Wood apple	*Aegle marmelos* (L.) Corrêa[2]	Fruit	Summer	Open space	Commonly consumed
166.*Damba*	Pomelo	*Citrus maxima* (Burm.) [4]	Fruit	Winter	Field (Goda)	Little consumed
167.*Taad Jo*	Palmyra fruit, ripe	*Borassus flabellifer* L.[1]	Fruit	Monsoon	Open space	Little consumed
168.*Siyali ka Beej/Lammah ka Beej*	NA	NA	Fruit	Spring	Forest	Little consumed
169.*Kandeyor*	NA	NA	Fruit	Spring	Forest	Little consumed
170.*Dola/Madkam*	Mahua fruit	*Madhuca longifolia* (J. Koenig. ex L.) J.F. Macbr. [2]	Fruit	Monsoon	Open space, forest	Commonly consumed
171.*Jangli suar/Bir sukri*	Wild pig	*Sus scrofa*[2]	Meat	Summer	Forest	Commonly consumed
172.*Hiran/Silip*	Deer	*Axis porcinus*[2]	Meat	Summer	Forest	Commonly consumed
173.*Kulhe/Khargosh*	Indian hare	*Lepus nigricollis*[10]	Meat	Winter, Summer	Forest	Little consumed
174.*Bhaid*	Sheep	*Ovis aries*[10]	Meat	Summer	Household rearing, Market	Little consumed
175.*Gilhari/tu*	Indian Palm Squirrel	*Funambulus palmarum*[2]	Meat	Summer	Forest	Little consumed
176.*Lomdi/tuyu*	Fox	*Vulpes vulpes*[2]	Meat	Winter	Forest	Little consumed

Local name	Common name	Scientific name	Part Consumed	Seasonality	Grown/Accessed	Food Preference
177.*Bakri*	Goat	*Capra aegagrus hircus*[10]	Meat	Spring, Summer	Household rearing, Market	Commonly consumed
178.*Bau*	Wild cat	NA	Meat	NA	Forest	Little consumed
179.*Tor*	Bengal monitor	*Varanus bengalensis* (Daudin, 1802) [4]	Meat	Spring, Summer	Forest	Little consumed
180.*Jackal/Siyar*	Jackal	*Canis aureus* Linnaeus, 1758[10]	Meat	Winter	Forest	Little consumed
181.*Musa/Guddu*	Field rat	*Rattus argentiventer*[2]	Meat	Autumn	Goda, Field (Pi)	Little consumed
182.*Jini/Jiki*	Porcupine	*Hystrix brachyura* Linnaeus, 1758[10]	Meat	NA	Forest	Historically consumed
183.*Oreh*	NA	NA	Meat	Summer	Forest	Little consumed
184.*Bana*	Bear	NA	Meat	Summer	Forest	Little consumed
185.*Putem (Bird)*	Spotted Dove	*Streptopelia chinensis*[2]	Meat	Summer	Open spaces, forest	Commonly consumed
186.*Jangli kabootar/Dudulum*	Pigeon	*Columba livia domestica*[2]	Meat	Summer	Forest	Commonly consumed
187.*Maina/Rami (Bird)*	Indian myna	*Acridotheres tristis*[2]	Meat	Summer	Open spaces, forest	Commonly consumed
188.*Dur/Durki/Bater (Bird)*	Quail	*Coturnix coturnix*[2]	Meat	Summer	Open spaces	Commonly consumed
189.*Mor*	Indian peafowl	*Pavo cristatus*[2]	Meat	Summer	Forest	Little consumed
190.*Pikodo/Tota/Mitthu (bird)*	Parrot	*Psittaciformes*[10]	Meat	All year	Forests	Little consumed
191.*Gauraiya/Dedem (Bird)*	House sparrow	*Passer domesticus* L. [10]	Meat	Summer	Open spaces, forest	Little consumed
192.*Bagula (Bird)*	Indian Heron	*Ardeola grayii*[10]	Meat	Summer	Open spaces	Little consumed
193.*Jangli Murga*	Wild hen	*Galloanserae* sp. [2]	Meat	Winter, Summer	Forests, household rearing	Commonly consumed
194.*Kidu (bird)*	NA	NA	Meat	Summer	Forests	Little consumed
195.*Dondu (bird)*	NA	NA	Meat	Annual	Forests	Little consumed

Local name	Common name	Scientific name	Part Consumed	Seasonality	Grown/Accessed	Food Preference
196.*Jerla*	NA	NA	Meat	All year	Trees	Little consumed
197.*Sidiyaim (Bird)*	NA	NA	Meat	Summer	Open spaces, forest	Little consumed
198.*Dauchodh (bird)*	NA	NA	Meat	Winter	Forests	Little consumed
199.*Huni (Bird)*	NA	NA	Meat	Winter, Spring	Forest	Little consumed
200.*Kokore (bird)/Owl*	Owl	*Strigiformes*[10]	Meat	Summer	Forests	Little consumed
201.*Desi Battak*	Duck	*Anas poecilorhyncha*[10]	Meat	Spring, Summer	Household rearing, Market	Commonly consumed
202.*Askal (Bird)*	NA	NA	Meat	Summer	Forest	Little consumed
203.*Gendadi bird*	NA	NA	Meat	Monsoon	Forests	Commonly consumed
204.*Magrayi/Magura*	Walking catfish	*Clarias batrachus*[2]	Meat	Monsoon	River	Commonly consumed
205.*Guneesa*	NA	NA	Meat	Monsoon	River	Commonly consumed
206.*Dandeeke fish*	Indian flying barb	*Esomus danricus* [4]	Meat	Monsoon	River	Commonly consumed
207.*Choda machli*	Spotted snakehead	*Channa punctata*[2]	Meat	Summer, Monsoon	River	Commonly consumed
208.*Bumbui machli*	Spotless spiny eel	*Macrognathus albus* (Hembrom 2021)[9]	Meat	Monsoon	River	Commonly consumed
209.*Genda*	Snail	*Pila Globosa*[2]	Meat	Summer, Monsoon	River, Field (Sal)	Commonly consumed
210.*Doodi machli*	NA	NA	Meat	Monsoon	River	Commonly consumed
211.*Koonche machli/Koocha Hayi*	Freshwater Mud eel	*Monopterus cuchia* (Ham,1822)[9]	Meat	Monsoon	River	Commonly consumed
212.*Chirpi/Haad machli/Pita Hayi*	Puti fish/Pool Barb	*Barbus* sp. [2]	Meat	Summer, Monsoon	River, Market	Commonly consumed
213.*Bayara machli*	NA	NA	Meat	Summer, Monsoon	River, Market	Commonly consumed

Local name	Common name	Scientific name	Part Consumed	Seasonality	Grown/Accessed	Food Preference
214.*Gadhayi machli*	Giant snakehead	*Channa marulius*, Ham,1822[11]	Meat	Monsoon	River	Commonly consumed
215.*Bova machli*	NA	NA	Meat	Monsoon	River, Market	Commonly consumed
216.*Bing Hayi*	NA	NA	Meat	All year	River	Commonly consumed
217.*Suli machli*	NA	NA	Meat	Summer	River	Commonly consumed
218.*Telpee machli*	Bele fish	*Glossogoboius giuris*[2]	Meat	Monsoon	River	Little consumed
219.*Dondo machli*	Freshwater eel	*Anguilla Anguilla*[1]	Meat	Summer, Monsoon	River	Commonly consumed
220.*Dele machli*	Eurasian carp	*Cyprinus carpio* Linnaeus, 1758 [4]	Meat	Monsoon	River	Little consumed
221.*Riba machli*	NA	NA	Meat	Monsoon	River	Commonly consumed
222.*Sisingke*	NA	NA	Meat	All year	River	Commonly consumed
223.*Choru machli*	NA	NA	Meat	Monsoon	River, Market	Commonly consumed
224.*Seep/Gechi*	Oyster	*Crassostrea madrasensis*[10]	Meat	All year	River	Commonly consumed
225.*Glashkar machli*	NA	NA	Meat	Winter	River	Little consumed
226.*Chauke/Mendak*	Frog	*Anura*[10]	Meat	Summer, Winter	River	Little consumed
227.*Horo*	Tortoise	*Testudinidae*[10]	Meat	Monsoon	River	Little consumed
228.*Kanta machli*	Bombay duck	*Harpadon nehereus*[1]	Meat	All year	River	NA
229.*Kurkut (Lal chiti)/Hauko*	Red ant	*Oecophylla smaragdina* Fabricius 1775[2]	Meat	Winter, Summer	Forest	Commonly consumed
230.*Surpain (Insect)*	NA	NA	Meat	Monsoon	Open spaces	Little consumed
231.*Madhumakkhi (larvae)*	Bee	*Apis mellifera* Linnaeus 1758[2]	Meat	Summer, Monsoon	Open spaces	Commonly consumed

Local name	Common name	Scientific name	Part Consumed	Seasonality	Grown/Accessed	Food Preference
232.*Bunum barui (bee)*	NA	NA	Meat	Summer	Open spaces	Little consumed
233.*Dumur (bee)*	NA	NA	Meat	Summer	Open spaces	Little consumed
234.*Lily (Juice of Lilyko)*	NA	*Apis mellifera*[2]	Meat	All year	Forest, Trees	Commonly consumed
235.*Burtud (insect)*	NA	NA	Meat	Monsoon	Open spaces	Little consumed
236.*Tumbli (Insect)*	Hornet & Wasp	*Vespa spp. / Ropalidia* spp. [2]	Meat	Summer	Open spaces	Little consumed
237.*Kolai (Insect)*	NA	NA	Meat	Summer	Forest	Commonly consumed
238.*Raipde (Insect)*	Girni	NA	Meat	Monsoon	Open spaces	Little consumed
239.*Hurum Suku(insect)*	NA	NA	Meat	Summer	Forests	Commonly consumed
240.*Kurkut (Lal chiti) eggs*	Eggs of red ant	*Oecophylla smaragdina* Fabricius 1775 [2]	Eggs	Winter, Summer	Forest	Commonly consumed
241.*Sidmi cheetu/Sem Keeda (Insect)*	NA	NA	Meat	Spring	Kitchen Garden, Field (Goda)	Commonly consumed
242.*Suttu/Terum (insect)*	NA	NA	Meat	Summer	Forests	Commonly consumed
243.*Rauum (Insect)*	NA	NA	Meat	Winter, Summer	Forest	Little consumed

NA, not available

[1]Longvah et al. 2017, [2] Ghosh-Jerath et al. 2021, [3] Ghosh-Jerath et al. 2016, [4] Ethnobotanist/Zoologist, [5] Ram & Sinha 2020, [6] Kumar 2015, [7]Ghosh-Jerath et al. 2020, [8]Manna et al. 2014, [9]Debnath et al. 2019, [10]India Biodiversity Portal 2022, [11]Hembrom 2021, [12(](Vishal et al., 2022), [13](Horo & Topno, 2015)

Supplementary methods

The taxonomic identification of listed indigenous foods (IFs) was done by reviewing previously published articles on IFs of Jharkhand (1–6) as well as the Indian Food Composition Table (IFCT) (7). Foods with no common names available in the secondary literature were taxonomically classified through photographs which were verified by a local ethnobotanist. The nutritive value of these identified IFs were searched in the Indian food composition database and other secondary literature and were collated. IFs with no secondary data on nutritional values, were collected from field sites using standard procedures developed as part of the larger study protocol and sent for analysis to a food testing laboratory accredited with National Accreditation Board for Testing and Calibration Laboratories (NABL). The parameters analyzed per 100g of edible food amount included energy, protein, carbohydrate, fat, dietary fiber, vitamin A (as beta-carotene), vitamin C, vitamin B1, B2, iron, calcium, zinc, folate, and phosphorous.

The entire data collection was conducted and supervised by the core research team. In addition to the core research team, the study team also included well-trained local field workers fluent in the native *Ho* dialect who assisted the team in facilitating the qualitative enquiries and collection of food samples for identification and nutritional analysis purposes. Two local field workers were given a two-day training prior to qualitative data collection wherein they were briefed about the study objectives and their role as translators during the qualitative enquiries. The two field workers were also trained regarding the steps of collection, packaging and transportation of food samples from the field to the ethnobotanist's laboratory and food analysis laboratory.
